# Association of sociodemographic factors and comorbidity with non-receipt of medications for secondary prevention: a cohort study of 12,204 myocardial infarction survivors

**DOI:** 10.1186/s12916-025-04160-5

**Published:** 2025-07-01

**Authors:** Ike Dhiah Rochmawati, Jocelyn M. Friday, Daniel Ang, Tran Q. B. Tran, Clea du Toit, Alan Stevenson, Jim Lewsey, Daniel Mackay, Ruth Dundas, Bhautesh Jani, S. Vittal Katikireddi, Christian Delles, Sandosh Padmanabhan, Carlos Celis-Morales, Paul Welsh, Frederick K. Ho, Jill P. Pell

**Affiliations:** 1 School of Health and Wellbeing, University of Glasgow, Glasgow, UK; 2 Department of Clinical and Community Pharmacy, Faculty of Pharmacy, University of Surabaya, Surabaya, Indonesia; 3 School of Cardiovascular and Metabolic Health, University of Glasgow, Glasgow, UK; 4 Human Performance Lab, Education, Physical Activity and Health Research Unit, University Católica del Maule, Talca, 3466706 Chile; 5 Centro de Investigacion en Medicina de Altura (CEIMA), Universidad Arturo Prat, Iquique, Chile

**Keywords:** Myocardial infarction, Medication, Comorbidity, Sociodemographic

## Abstract

**Background:**

Clinical guidelines recommend use of (1) antiplatelet, (2) lipid-lowering, and (3) beta blocker medication, and (4) angiotensin-converting enzyme inhibitor or angiotensin receptor blocker (ACEi/ARB) for secondary prevention following myocardial infarction (MI). This study examines whether sociodemographic factors and comorbidity were associated with receipt of guideline-recommended medication, and whether receipt was associated with all-cause mortality.

**Methods:**

A cohort study was conducted on West of Scotland patients aged 53 years or above who were discharged from hospital alive after an incident MI between 2014 and 2022. Receipt of guideline-directed therapy was defined as relevant medications dispensed within 3 months of discharge. Age, sex, area-deprivation, care/nursing home residence, year of incident MI, and pre-existing conditions were included as predictors of non-receipt and covariates in the analysis of the association between non-receipt and death.

**Results:**

Among 12,204 MI survivors, 7898 (64.72%) received all four classes of recommended medications. Non-receipt increased over the study period and was more likely in women, older people, more deprived people, care/nursing home residents, or those with preexisting atrial fibrillation, chronic kidney disease, liver diseases, chronic obstructive pulmonary disease, or psychosis; and was less likely in those who had prior revascularisation. Non-receipt was associated with higher mortality (HR 1.15, 95% CI 1.05–1.26) after adjusting for sociodemographic factors and preexisting conditions. Excess mortality due to area deprivation and care/nursing home residence could be partly explained by non-receipt of ACEi/ARB (9.4% for deprivation; 40.7% for care/nursing home residence) and lipid lowering medication (39.7% for care/nursing home residence).

**Conclusions:**

Recommended secondary prevention medications were less likely to be received by women, those deprived, living in care/nursing homes, and with comorbid conditions. Equivalising appropriate ACEi/ARB use for secondary prevention could slightly reduce socioeconomic inequality of cardiovascular mortality.

**Supplementary Information:**

The online version contains supplementary material available at 10.1186/s12916-025-04160-5.

## Background

Following myocardial infarction (MI), patients have a high risk of recurrent cardiovascular events and death [1]. Secondary prevention in these patients requires multiple components, including the use of medications [2]. Clinical guidelines [3, 4] recommend antiplatelet, beta blocker (BB), angiotensin converting enzyme inhibitor (ACEi), and lipid-lowering medications following MI, unless contraindicated, as they have been shown to reduce major adverse cardiovascular events in clinical trials [5, 6]. Nonetheless, the proportion of people receiving secondary prevention medication, as recommended by guidelines, has been found to be low, irrespective of drug class [7]. In a previous study, following treatment guidelines was associated with lower 1-year mortality following acute coronary syndromes (ACS), but was only achieved by 40% of patients [8]. Factors shown to be associated with guideline-directed prescribing have included comorbidities (chronic lung disease, peripheral artery disease (PAD), and renal impairment), MI classification, and the administration of reperfusion therapy [9]. Failure to prescribe medication can be due to real or perceived contraindications. For example, beta blockers were historically contraindicated for patients with chronic obstructive pulmonary disease [10] but, whilst modern therapies have been shown to be safe, they remain under-prescribed [11]. Conversely, certain diagnoses or treatments may increase adherence to guideline-recommended prescribing; such as beta blockers being initiated to treat hypertension prior to the MI. However, whether, and to which extent, variations in receipt of recommended treatment are associated with mortality has not been reported.

In this study, we assessed whether sociodemographic and comorbid factors were associated with receipt of recommended secondary prevention medication among MI survivors, and whether that was, in turn, associated with all-cause mortality, overall and by comorbidity subgroup.

## Methods

### Study design

A cohort study was constructed using linked extracts of electronic health records of patients treated in NHS Greater Glasgow and Clyde. The data were held and analysed in the West of Scotland Safe Haven, a trusted research environment. The study sample included patients aged 53 years or above who experienced an incident MI in 2014–2022 and were discharged alive after the event. A 2-year lookback period was applied to identify incident MIs. Data were available for residents aged 51 or above in 2012 and therefore this study included only individuals aged 53 or above in 2014 to accommodate the lookback period. Data on dispensed medications prescribed in the community [12] were used to ascertain adherence to the guidelines within the first 3 months after discharge from the index event. Patients who died within 3 months after discharge were excluded. Follow-up for mortality events start from 3 months after discharge.

### Measurements

This study used linked data on general hospital admissions (Scottish Morbidity Record 01 (SMR01)), psychiatric hospital admissions (Scottish Morbidity Record 04 (SMR04)), death certificates, and dispensed prescriptions (Prescription Information System).

MIs were ascertained from SMR01 records with a relevant ICD-10 code (Additional File 1: Table S1) in any diagnostic position between 1 January 2014 and 31 December 2022. To only include incident acute MIs, patients who had an admission with MI within the previous 2 years were excluded, and only the first MI event over the study period was included. People who died in-hospital during their MI admission were also excluded.

The primary outcome was receipt of medications for secondary prevention, as recommended by the UK NICE (National Institute for Health and Care Excellence), European Society of Cardiology, and American Heart Association [3–5, 13]; specifically, at least one dispensed prescription for each of the recommended drug classes over the three months from hospital discharge following incident MI. These were defined as: antiplatelet therapy, lipid lowering agents, beta blockers (or two specific calcium channel blockers (CCB) if beta blockers were contraindicated), ACEi (or angiotensin-II receptor blocker, ARB and ARNI (angiotensin receptor-neprilysin inhibitor)). The BNF (British National Formulary) codes for these are listed in Additional File 1: Table S1. The secondary outcome was all-cause mortality among people discharged alive from hospital following MI.

Sociodemographic covariates included area deprivation (using the Scottish Index of Multiple Deprivation (SIMD) quintile 2012), age at incident MI, sex, and care/nursing home residence, included assisted living as well as those requiring skilled nursing care. Care/nursing home residence was ascertained as any care/nursing home dispensing in the Prescription Information System within 2 years prior to the index event.

Preexisting comorbidity covariates included angina, coronary revascularisation (elective percutaneous coronary intervention [PCI] or coronary artery bypass grafting [CABG]), peripheral artery disease (PAD), abdominal aortic aneurysm (AAA), heart failure (HF), diabetes, chronic kidney disease (CKD), liver disease, chronic obstructive pulmonary disease (COPD), psychosis, and depression, ascertained from relevant diagnostic, procedure, and prescription codes within 2 years prior to the index MI event. The methods and codes to ascertain these are shown in Additional file 1: Table S1.

### Statistical analyses

Non-receipt of guideline-directed medication was analysed using binary logistic regression, with age, sex, SIMD quintile, care/nursing home residence, and comorbid conditions included as predictors in two incremental models: Model 1 included age, sex, and SIMD quintile only; Model 2 additionally included care/nursing home residence and comorbid conditions. Because care/nursing home residence and comorbid conditions could be the causal sequelae of other sociodemographic factors, Model 1 was used to derive the total association for sociodemographic factors.

Cox proportional hazard models were used to estimate the association between non-receipt and all-cause mortality using hazard ratios (HR) and their 95% confidence intervals, adjusted for age, sex, and SIMD, and weighted using fine stratification weights derived from the propensity score for non-receipt [14]. SIMD quintiles were used as a numeric variable scaled from 0 (least deprived) to 1 (most deprived) so that the coefficients could be interpreted as the relative index of inequality (RII) [15]. The propensity score was derived using a binary logistic model of age, sex, year, care/nursing home residence, and all included comorbid conditions. Fifty strata were created based on patients who did not receive the medications (exposed group), which was shown to be robust in most situations [16]. Subgroup analyses were conducted by comorbidity subgroups (angina and revascularisation, heart failure, other CVDs, chronic obstructive pulmonary disease, diabetes, chronic kidney disease, and psychosis and depression) using the same adjustment model and similarly constructed regression weights. Because medication receipt was defined as within three months following hospital discharge, we conducted a landmark analysis in which follow-up for mortality started from 3 months after discharge to avoid immortal time bias.

In order to quantify the extent to which non-receipt of medication could explain the excess risk associated with area deprivation and care/nursing home residence, we also conducted a mediation analyses [17] based on g-formula, with 2000 bootstrapping for 95% CIs. The mediation analyses were conducted based on the causal hypothesis shown in Additional file 2: Fig. S1. The exposure variables were area deprivation and care/nursing home residence, non-receipt of the medications was the mediator, and mortality was the outcome. Comorbidities were adjusted as post-exposure (deprivation and care/nursing home residence) mediator-outcome confounders. Statistical analyses were performed using R Statistical Package version 4.3.0 with the *survival* and *CMAverse* packages. 

### Ethics and data extraction methods

Delegated research ethics approval was granted for linkage to National Health Service (NHS) patient data by the Local Privacy and Advisory Committee at NHS Greater Glasgow and Clyde.

Cohorts and de-identified linked data were prepared by the West of Scotland Safe Haven Research Database at NHS Greater Glasgow and Clyde (IRAS Project ID 321198, REC reference 22/WS/1063). The West of Scotland Safe Haven is a partnership between the University of Glasgow and NHS Greater Glasgow and Clyde to provide secure access to NHS health datasets relating to citizens in the region. More details can be found here: https://www.nhsggc.scot/hospitals-services/services-a-to-z/west-of-scotland-safe-haven/. The study’s STROBE checklist is shown in Additional file 3.

## Results

There were 14,757 incident MI events from 2014, of which 12,857 were discharged alive. After excluding 27 patients who died within 3 months of discharge, the study population comprised 12,204 patients. Overall, 7898 (64.72%) patients received medications consistent with the guidelines for secondary prevention following acute MI [3–5, 13]. Patients who did not receive all the recommended medications were older, more likely to live in care/nursing homes, more likely to have had a stroke, PAD, AF, AAA, HF, CKD, psychosis, and depression, but less likely to have had angina or coronary revascularization previously (Table 1). Of the four classes of recommended medications, ACEi/ARB (37.2%) were dispensed least frequently, followed by BB/CCB (55.4%), lipid-lowering medications (66.2%), then antiplatelets (79.3%).

**Table 1:** Patient characteristics by receipt of recommended medications following discharge from myocardial infarction

	**Overall**	**Receipt of all recommended medications**	
		**No**	**Yes**
	*N* = 12,204	*N* = 4306	*N* = 7898
Mean (SD) age in years at MI	66.30 (9.99)	70.37 (10.52)	64.08 (8.94)
Sex			
Female	5232 (42.9%)	2172 (50.4%)	3060 (38.7%)
Male	6972 (57.1%)	2134 (49.6%)	4838 (61.3%)
Care/nursing home residence	919 (7.5%)	605 (14.1%)	314 (4.0%)
Year of incident MI			
2014–2016	4777 (39.1%)	1783 (41.4%)	2994 (37.9%)
2017–2019	4111 (33.7%)	1456 (33.8%)	2655 (33.6%)
2020–2022	3316 (27.2%)	1067 (24.8%)	2249 (28.5%)
Pre-existing conditions			
Angina	3172 (26.0%)	1031 (23.9%)	2141 (27.1%)
CABG	768 (6.3%)	214 (5.0%)	554 (7.0%)
PCI	6111 (50.1%)	1191 (27.7%)	4920 (62.3%)
Stroke	1571 (12.9%)	763 (17.7%)	808 (10.2%)
PAD	1230 (10.1%)	515 (12.0%)	715 (9.1%)
AF	3193 (26.2%)	1503 (34.9%)	1690 (21.4%)
AAA	298 (2.4%)	144 (3.3%)	154 (1.9%)
HF	5888 (48.2%)	2555 (59.3%)	3333 (42.2%)
Diabetes	3660 (30.0%)	1330 (30.9%)	2330 (29.5%)
CKD	2469 (20.2%)	1319 (30.6%)	1150 (14.6%)
Liver disease	517 (4.2%)	224 (5.2%)	293 (3.7%)
COPD	5009 (41.0%)	2015 (46.8%)	2994 (37.9%)
Psychosis	781 (6.4%)	436 (10.1%)	345 (4.4%)
Depression	6029 (49.4%)	2258 (52.4%)	3771 (47.7%)
Post MI therapy			
CABG	249 (2.0%)	53 (1.2%)	196 (2.5%)
PCI	830 (6.8%)	210 (4.9%)	620 (7.9%)
Antiplatelets	11,312 (92.7%)	3414 (79.3%)	7898 (100%)
Lipid lowering medications	10,748 (88.1%)	2850 (66.2%)	7898 (100%)
ACEi/ARB	9499 (77.8%)	1601 (37.2%)	7898 (100%)
BB/CCB	10,284 (84.3%)	2386 (55.4%)	7898 (100%)

*AAA* abdominal aortic aneurysm, *ACEi* angiotensin converting enzyme inhibitor, *AF* atrial fibrillation, *ARB* angiotensin receptor blocker, *BB* beta blocker, *CABG* coronary artery bypass graft, *CCB* calcium channel blocker, *CKD* chronic kidney disease, *COPD* chronic obstructive airways disease, *HF* heart failure, *MI* myocardial infarction, *N* number, *PAD* peripheral artery disease, *PCI* percutaneous coronary intervention, *SD* standard deviation, *SIMD* Scottish Index of Multiple Deprivation

In Model 1, older age, female sex, and higher socioeconomic deprivation were associated with not meeting the guidelines overall, with all four of the drug classes being less likely to be taken by older individuals (Table 2). The association with deprivation was substantially attenuated after adjustment for comorbidities (Model 2 OR 1.00, 95% CI 0.89–1.12). After adjusting for sociodemographic factors, non-receipt of medications was associated with care/nursing home residence (OR 1.65, 95% CI 1.40–1.94), AF (OR 1.28; 95% CI 1.16–1.41), AAA (OR 1.46; 95% CI 1.13–1.88), CKD (OR 1.64; 95% CI 1.48–1.82), liver disease (OR 1.43; 95% CI 1.18–1.74), COPD (OR 1.34; 95% CI 1.23–1.46), and psychosis (OR 1.42; 95% CI 1.20–1.67). Conversely, patients who had undergone PCI (OR 0.31; 95% CI 0.29–0.34) and CABG (OR 0.59; 95% CI 0.49–0.70) prior to the index MI were more likely to receive all recommended medications.

**Table 2:** Logistic regression analyses of the associations between sociodemographic factors and preexisting conditions and non-receipt of medications

	**All four classes**		**Antiplatelet**		**Lipid-lowering**		**ACEi/ARB**		**BB/CCB**	
	**OR**	**95% CI**	**OR**	**95% CI**	**OR**	**95% CI**	**OR**	**95% CI**	**OR**	**95% CI**
**Model 1**										
Age	1.07	1.06–1.07	1.04	1.04–1.05	1.07	1.06–1.07	1.06	1.06–1.07	1.05	1.04–1.05
Male sex	0.83	0.77–0.90	0.89	0.77–1.02	0.81	0.72–0.91	0.83	0.76–0.91	1.02	0.92–1.13
Year of MI	1.03	1.01–1.04	1.01	0.99–1.04	1.01	0.99–1.03	1.02	1.00–1.04	1.03	1.01–1.05
SIMD (RII)	1.25	1.12–1.38	1.06	0.88–1.27	1.04	0.90–1.21	1.33	1.18–1.50	1.18	1.03–1.34
**Model 2**										
Age	1.04	1.03, 1.04	1.00	0.99, 1.01	1.04	1.03, 1.04	1.03	1.03, 1.04	1.03	1.03, 1.04
Male sex	1.01	0.92, 1.10	1.04	0.90, 1.21	0.95	0.84, 1.07	0.96	0.87, 1.06	1.19	1.07, 1.33
Year of MI	1.03	1.01, 1.05	1.01	0.98, 1.04	1.01	0.99, 1.03	1.03	1.01, 1.05	1.03	1.01, 1.05
SIMD (RII)	1.00	0.89, 1.12	0.89	0.73, 1.08	0.92	0.79, 1.08	1.11	0.98, 1.26	1.03	0.90, 1.19
Care/nursing home	1.65	1.40–1.94	1.59	1.28–1.98	1.56	1.31–1.86	1.73	1.48–2.03	1.50	1.27–1.78
Angina	0.94	0.85–1.03	0.77	0.65–0.92	0.81	0.70–0.94	0.99	0.89–1.10	0.87	0.77–0.98
CABG	0.59	0.49–0.70	0.35	0.24–0.50	0.45	0.33–0.61	0.88	0.72–1.07	0.49	0.37–0.63
PCI	0.31	0.29–0.34	0.16	0.13–0.20	0.27	0.24–0.32	0.32	0.29–0.36	0.44	0.39–0.49
Stroke	1.12	0.99–1.26	0.91	0.74–1.10	0.91	0.78–1.07	1.17	1.02–1.32	1.06	0.92–1.23
PAD	1.18	1.03–1.35	0.86	0.67–1.09	1.20	0.99–1.44	1.33	1.15–1.53	0.96	0.81–1.14
AF	1.28	1.16–1.41	2.42	2.08–2.83	1.15	1.01–1.30	1.18	1.06–1.31	0.85	0.75–0.96
AAA	1.46	1.13–1.88	1.39	0.91–2.04	1.01	0.70–1.42	1.40	1.06–1.82	1.27	0.94–1.70
HF	1.00	0.91–1.10	0.96	0.81–1.13	1.04	0.91–1.19	0.88	0.79–0.98	0.84	0.75–0.95
Diabetes	0.93	0.85–1.02	1.01	0.86–1.19	0.85	0.74–0.97	0.96	0.86–1.06	0.97	0.87–1.09
CKD	1.64	1.48–1.82	1.13	0.96–1.34	1.20	1.05–1.38	1.87	1.68–2.09	0.98	0.86–1.11
Liver disease	1.43	1.18–1.74	1.27	0.92–1.72	1.68	1.30–2.16	1.18	0.95–1.47	1.20	0.94–1.52
COPD	1.34	1.23–1.46	0.94	0.81–1.09	0.98	0.87–1.11	1.17	1.07–1.29	1.48	1.33–1.64
Psychosis	1.42	1.20–1.67	1.13	0.88–1.44	1.65	1.37–2.00	1.61	1.36–1.90	1.10	0.91–1.32
Depression	1.03	0.94–1.12	0.88	0.76–1.02	0.88	0.78–1.00	1.05	0.95–1.16	1.02	0.91–1.13

All variables were mutually adjusted within the model

*AAA* abdominal aortic aneurysm, *AF* atrial fibrillation, *CABG* coronary artery bypass graft, *CI* confidence interval, *CKD* chronic kidney disease, *COPD* chronic obstructive pulmonary disease, *HF* heart failure, *OR* odds ratio, *PAD* peripheral artery disease, *PCI* percutaneous coronary intervention, *SIMD (RII)* Relative index of inequality using Scottish Index of Multiple Deprivation

Comorbid conditions were associated with the non-receipt of medications, as detailed in Table 2. AF (OR 2.42; 95% CI 2.08–2.83) and COPD (OR 1.48, 95% CI 1.33–1.64) were most strongly associated with the non-receipt of antiplatelet agents and beta-blockers, respectively. Liver disease (OR 1.68; 95% CI 1.30–2.16) and psychosis (OR 1.65; 95% CI 1.37–2.00) were most linked to the non-receipt of lipid-lowering medication. Additionally, CKD (OR 1.87, 95% CI 1.68–2.09) and psychosis (OR 1.61; 95% CI 1.36–1.90) were most strongly associated with the non-receipt of angiotensin-converting enzyme ACEi/ARB.

The associations between non-receipt of medications and all-cause mortality are shown in Table 3. Non-receipt of at least one of the drug classes was associated with higher mortality risk overall (HR 1.15; 95% CI 1.05–1.26), and among patients with HF (HR 1.30; 95% CI 1.17–1.45), other CVDs, including stroke, AF, PAD, and AAA (HR 1.18; 95% CI 1.05–1.32), CKD (HR 1.43; 95% CI 1.24–1.65), and psychosis/depression (HR 1.13; 95% CI 1.00–1.28). The association was weaker in the subgroup of people with a history of angina/coronary revascularisation (HR 1.11; 95% CI 0.98–1.27), COPD (HR 1.12; 95% CI 0.98–1.28), and diabetes (HR 1.16; 95% CI 1.00–1.36).

**Table 3:** Cox proportional hazard models of the associations between non-receipt of medications and all-cause mortality, overall and by comorbidity subgroup

	**Any of the four classes**		**Antiplatelet**		**Lipid lowering**		**ACEi/ARB**		**BB/CCB**	
	**HR**	**95% CI**	**HR**	**95% CI**	**HR**	**95% CI**	**HR**	**95% CI**	**HR**	**95% CI**
Overall	1.15	1.05, 1.26	1.04	0.94, 1.15	1.24	1.14, 1.34	1.23	1.15, 1.32	1.09	1.01, 1.17
Comorbidity subgroup										
Angina + revascularisation	1.11	0.98, 1.27	1.19	0.97, 1.47	1.19	1.02, 1.39	1.13	1.00, 1.27	1.18	1.04, 1.34
HF	1.30	1.17, 1.45	1.10	0.98, 1.24	1.29	1.17, 1.42	1.35	1.25, 1.47	1.14	1.04, 1.25
Other CVDs	1.18	1.05, 1.32	1.03	0.92, 1.17	1.19	1.07, 1.32	1.28	1.17, 1.40	1.10	0.99, 1.21
COPD	1.12	0.98, 1.28	1.24	1.07, 1.44	1.37	1.22, 1.55	1.30	1.18, 1.44	1.14	1.02, 1.26
Diabetes	1.16	1.00, 1.36	1.04	0.87, 1.24	1.02	0.88, 1.18	1.27	1.13, 1.43	1.03	0.90, 1.17
CKD	1.43	1.24, 1.65	0.98	0.83, 1.16	1.23	1.08, 1.40	1.47	1.32, 1.65	1.13	0.99, 1.28
Psychosis and depression	1.13	1.00, 1.28	1.07	0.93, 1.23	1.31	1.18, 1.46	1.19	1.08, 1.30	1.18	1.07, 1.31

Analysis was weighted using fine propensity score fine stratification weights; propensity score was derived from sociodemographic factors and all comorbid conditions

*HR* hazard ratio, *CI* confidence interval, *CKD* chronic kidney disease, *COPD* chronic obstructive pulmonary disease, *CVD* cardiovascular diseas

Of each of the individual drug classes, non-receipt of lipid lower medications was associated with the highest mortality risk (HR 1.24; 95% CI 1.14–1.34), followed by ACEi/ARB (HR 1.23; 95% CI 1.15–1.32), and BB/CCB (HR 1.09; 95% CI 1.01–1.17). The association with non-receipt of antiplatelet medication was weaker (HR 1.04; 95% CI 0.94–1.15).

Non-receipt of lipid lowering medications was associated with highest mortality risk among patients with COPD (HR 1.37; 95% CI 1.22–1.55), psychosis or depression (HR 1.31; 95% CI 1.18–1.46), HF (1.29; 95% CI 1.07–1.32), other CVDs (HR 1.19; 1.07–1.32), and angina and prior coronary revascularisation (HR 1.19; 95% CI 1.02–1.39). The association was weak in the subgroup with diabetes (HR 1.02; 95% CI 0.88–1.18).

Non-receipt of ACEi/ARB was associated with mortality in all subgroups. The strongest association was in people with CKD (HR 1.47; 95% CI 1.32–1.65), followed by HF (HR 1.35; 95% CI 1.25–1.47), COPD (HR 1.30; 95% CI 1.18–1.44), other CVDs (1.28; 95% CI 1.17–1.40), diabetes (HR 1.27; 95% CI 1.13–1.43), psychosis and depression (HR 1.19; 95% CI 1.08–1.30), then angina and previous coronary revascularisation (HR 1.13; 95% CI 1.00–1.27).

Non-receipt of BB/CCB was associated with mortality in people with a history of angina and coronary revascularisation (HR 1.18; 95% CI 1.04–1.34), psychosis and depression (HR 1.18; 95% CI 1.07–1.31), HF (HR 1.14; 95% CI 1.04–1.25), and COPD (HR 1.14; 95% CI 1.02–1.26). The association was weaker in the subgroup with other CVDs (HR 1.10; 95% CI 0.99–1.21), diabetes (HR 1.03; 95% CI 0.90–1.17), and CKD (HR 1.18; 95% CI 1.07–1.31). Non-receipt of antiplatelet therapy was only associated with mortality in patients with COPD (HR 1.24; 95% CI 1.07–1.44).

Table 4 shows the extent to which non-receipt of medication could explain the excess mortality risk attributed to area deprivation and care/nursing home residence. The model accounted for post-exposure confounding due to comorbidity and confirmed associations between SIMD (HR 1.37; 95% CI 1.24–1.48) and care/nursing home residence (HR 2.46; 95% CI 2.25–2.67) with mortality. A small proportion (9.4%) of excess mortality risk attributed to area deprivation could be explained by non-receipt of ACEi/ARB. However, non-receipt of at least one of the drug class explained almost 40% of excess risk in care/nursing home residence; the majority of which was due to non-receipt of lipid lowering medications and/or ACEi/ARB.

**Table 4:** Non-receipt of guideline-directed medications as a potential mechanism for the mortality inequality by SIMD or care/nursing home residence

	**All classes**		**Antiplatelet**		**Lipid lowering**		**ACEi/ARB**		**BB/CCB**	
	Estimate	(95% CI)	Estimate	(95% CI)	Estimate	(95% CI)	Estimate	(95% CI)	Estimate	95% CI
**SIMD (RII)**										
Natural direct effect	1.34	(1.24, 1.48)	1.36	(1.26, 1.48)	1.41	(1.3, 1.57)	1.35	(1.24, 1.48)	1.34	(1.23, 1.45)
Natural indirect effect	1.02	(0.99, 1.05)	1.00	(0.98, 1.01)	0.99	(0.97, 1.02)	1.03	(1.01, 1.06)	1.02	(0.99, 1.03)
Total effect	1.37	(1.25, 1.51)	1.36	(1.25, 1.48)	1.40	(1.3, 1.55)	1.38	(1.27, 1.53)	1.36	(1.25, 1.46)
% mediated	6.64	(− 2.19, 16.07)	− 0.31	(− 7.15, 4.69)	− 5.03	(− 12.15, 6.20)	9.43	(2.96, 21.37)	5.73	(− 3.59, 9.11)
**Care/nursing home residence**										
Total effect	2.46	(2.25–2.67)	2.36	(2.16–2.59)	2.37	(2.19–2.62)	2.25	(2.06–2.48)	2.37	(2.14–2.58)
Natural direct effect	1.88	(1.73–2.05)	2.13	(1.97–2.31)	1.83	(1.68–2.03)	1.74	(1.59–1.90)	2.08	(1.88–2.26)
Natural indirect effect	1.31	(1.25–1.34)	1.11	(1.08–1.14)	1.30	(1.25–1.36)	1.29	(1.24–1.34)	1.14	(1.10–1.17)
% mediated	39.5	(33.9–44.0)	16.7	(12.1–21.3)	39.7	(34.2–45.6)	40.7	(35.4–46.8)	21.2	(16.4–24.8)

Estimates shown are hazard ratio for % mediated. *SIMD (RII)* Relative index of inequality using Scottish Index of Multiple Deprivation

## Discussion

This study demonstrated that sociodemographic factors and comorbidity were associated with non-receipt of guideline-directed medications following MI, which, in turn, was associated with case fatality after adjusting for sociodemographic and comorbid factors. These inequalities compound the known differences in risk of incident MI by age, socioeconomic status, and comorbidity. We also found the excess risk associated with deprivation could be explained partly by non-receipt of ACEi/ARB, and that associated with care/nursing home residence could be explained by non-receipt of ACEi/ARB and lipid lowering medications.

Alongside lifestyle modification, using guideline-recommended medical therapy is an important component of secondary prevention, which, consistent with the present findings, has been shown to reduce the risk of major adverse cardiovascular events following MI [18]. This study found that two-thirds of patients did not receive all medications recommended by guidelines following MI. This finding is consistent with those reported in other countries such as Germany (68.61%) [19], Australia (65%) [20], and the USA (69%) [21].

### Age and care/nursing home as a predictor of medication receipt

Inequality was observed in the prescription of secondary prevention medications for cardiovascular disease, despite clinical guidelines recommending these medications for all patients other than those with contraindications. Our study identified that older patients, and those living in a care/nursing home, had a lower likelihood of receiving guideline-recommended medications. These findings are consistent with, but extend, those of previous studies [22–24]. A potential reason for the reduced likelihood of older patients receiving all recommended medications is that physicians often prescribe fewer drugs due to age-related declines in pharmacokinetic parameters, such as absorption, distribution, metabolism, and elimination. Despite clinical guidelines recommending evidence-based secondary preventive medications for MI across all age groups, younger patients were more frequently prescribed these therapies upon discharge [23–26]. In the context of non-receipt of guideline-directed medication among care/nursing home residents, it is essential to recognise that the appropriateness of prescribing medications is based on specific clinical circumstances and individual patient needs. This study does not have data on whether care decisions were explicitly made as part of the patient's treatment plan for reasons not being captured in the routine data. One particular example is that some patients might be in palliative care where initiation of secondary prevention is not appropriate. Further investigation is required to assess the decision-making processes and ethical frameworks guiding medication management in care home settings. Indeed, there is a crucial need to balance risks and benefits in secondary prevention strategies among older people in both care homes and communities. There are existing tools for this purpose, e.g., STOPP (Screening Tool of Order People’s Potentially Inappropriate Prescriptions) and START (Screening Tool to Alert Doctors to Right Treatments) [27].

### Sex differences in medication receipt

Although clinical guidelines recommend the same treatment for both male and female patients, their real-world application often diverges. The findings on sex differences in receiving medications are also consistent with previous studies [28–30, 32]. Another study highlighted disparities in the administration of evidence-based medications for MI, with women being less likely to receive appropriate treatment due to an underestimation of their cardiovascular risk. These disparities arise from a complex interplay of biological differences and implicit biases in clinical decision-making [29, 33].

### Socioeconomic determinants of medication receipt

One study showed that patients in the lowest socioeconomic strata were less likely to receive secondary prevention pharmacotherapy and to achieve the treatment goals, attributing that to cost-related issues [31]. Since prescriptions are free in Scotland, cost should not have been a barrier to access those medications. Instead, since the associations between SIMD and non-receipt were attenuated and became non-significant after adjusting for comorbidities, our findings suggest that comorbidities might be a primary reason for not receiving medication in some patients.

### Comorbidity and medication receipt

Broadly consistent with previous studies, our findings showed that patients with most comorbid conditions were less likely to receive guideline-recommended medications following MI. The non-receipt of medications in patients with comorbidities could be due to patients with these comorbidities being less represented in clinical trials, casting worries on whether the medications would be beneficial to them. For example, there are concerns in CKD patients about elevated creatinine concentrations [34, 35, 40], and platelet dysfunction associated with uraemia [36], and in liver disease patients about biotransformation and drug clearance [37, 41–43]. However, consistent with this study’s finding, the administration of guideline-directed medications in haemodialysis patients was associated with a reduced risk of mortality [38], and a recent study found that, in patients with chronic liver disease and atherosclerotic cardiovascular disease, higher intensity statin therapy was associated with reduced all-cause mortality [39]. Apart from that, the non-receipt of guideline-directed medication could also be related to less up-to-date knowledge on contraindications. For example, BB was thought to be contraindicated in COPD patients even though cardioselective BB was later shown to not produce adverse respiratory effects [11].

There could be other reasons why psychosis patients were less likely to receive guideline-directed medications [44], e.g. due to the stigma associated with mental health problems, lack of insurance especially in socioeconomically deprived areas, less frequent medical contact, and physical and mental limitations from comorbid conditions [46]. The treatment gap between patients with psychosis and the general population can be minimized using multidisciplinary care models with input from psychiatrists, general physicians, and cardiologists [45].

### Strengths and limitations of this study

This large-scale, non-selective study included all patients experiencing an incident MI in the study population. We used propensity score fine stratification methods based on sociodemographic factors and comorbidity to reduce confounding. However, as with all observational studies, residual confounding cannot be ruled out. Receipt of the medications was ascertained through dispensed medications, but we cannot be certain that patients took the medications after collecting them. We were also unable to ascertain whether the failure to dispense medication was due to doctors not issuing prescriptions or patients not cashing the prescriptions. Longitudinal receipt of medication and its time-varying association with mortality were not studied. Information on reasons for contraindications or drug intolerance to secondary preventive medications that led to non-receipt and/or cessation was not available.

### Clinical implications

Clinical guidelines recommend pharmaceutical secondary prevention following MI, but our study showed that a sizable proportion of patients did not receive these medications, which was largely driven by comorbidities but is unlikely to be fully explained by legitimate contraindications. The National Institute for Health and Care Excellence (NICE) guidelines provide alternatives for patients with contraindications to certain medications. The integration of a simplified algorithm into routine clinical practice could help improve the coverage of guideline-recommended medication.

## Conclusions

Established inequalities in the risk of experiencing MI are compounded by subsequent inequalities in receipt of secondary prevention and, thereby, mortality. Recommended secondary prevention medications were less likely to be received by those deprived, living in care/nursing homes, and with comorbid conditions even if those are not contraindications. This could modestly explain the excess post-MI mortality in care/nursing homes and in areas with higher deprivation.

## Supplementary Information

Additional file 1: Table S1 – Ascertainment of conditions.

Additional file 2: Figure S1 – Causal assumptions of this study.

Additional file 3: STROBE checklist.

## Data Availability

No datasets were generated or analysed during the current study.

## Abbreviations

AAA: Abdominal aortic aneurysm
ACEI: Angiotensin converting enzyme inhibitor
ACS: Acute coronary syndromes
ARB: Angiotensin receptor blocker
ARNI: Angiotensin receptor-neprilysin inhibitor
BB: Beta blocker
BNF: British National Formulary
CABG: Coronary artery bypass graft
CCB: Calcium channel blocker
CKD: Chronic kidney disease
COPD: Chronic obstructive pulmonary disease
HF: Heart failure
HR: Hazard ratio
ICD: International Classification of Disease
MI: Myocardial infarction
NHS: National Health Service
NICE: National Institute for Health and Care Excellence
PAD: Peripheral artery disease
PCI: Percutaneous coronary intervention
SIMD: Scottish Index of Multiple Deprivation
SMR: Scottish Morbidity Report
STOPP: Screening Tool of Order People’s Potentially Inappropriate Prescriptions
START: Screening Tool to Alert Doctors to Right Treatments
