# Supplementary material for: Association of sociodemographic factors and comorbidity with non-receipt of medications for secondary prevention: a cohort study of 12,204 myocardial infarction survivors
Source: BMC Med. 2025 Jul 1;23:381. doi: 10.1186/s12916-025-04160-5 (PMC12219715; doi:10.1186/s12916-025-04160-5)
Supplement: Supplementary file 1 — Additional file 1: Table S1 – Ascertainment of conditions. [file 12916_2025_4160_MOESM1_ESM.docx]

Supplementary Table 1. Ascertainment of conditions

|  | **ICD-10 codes** | **OPSC-4 codes** | **BNF codes** |
| --- | --- | --- | --- |
| **Medical conditions** |  |  |  |
| Myocardial infarction | I21 | K502  K503 | - |
| Angina | I20 | - | - |
| CABG | - | K40  K41  K42  K43  K440  K441  K442  K448  K449  K450  K451  K452  K453  K454  K455  K456  K458  K459  K460  K461  K462  K464  K465  K468  K469 | - |
| PCI | - | K471  K49  K500  K501  K504  K508  K509  K75 | - |
| Hypertension | I10  I11  I12  I13  I15 | X828  X829 | 020201  020203  020502  020504  0205051  0205052 |
| Stroke | G463  G464  G465  G466  G467  I64  I672  I679  I691  I692  I694  I698  I63  I693 | U543  L294  L295  L296  L297  L311  L314  L353  L372  I60  I61  I620  I621  I629  I690 | - |
| PAD | I731  I738  I739  I743  I744  I745 | L50  L51  L52  L530  L531  L532  L541  L542  L544  L548  L549  L58  L59  L60  L620  L621  L622  L628  L629  L631  L632  L633  L635  L650  L651  L652  L653 |  |
| AF/AFL | I48 | - | - |
| AAA | I713  I714  I715  I716  I718  I719 | - | - |
| HF | I50  I420  I429  I110  I132  I130 | - | 020202 |
| Diabetes | E10-14 | - | 060101  060102 |
| CKD | N18 | - | - |
| Liver disease | K70-K77 | - | - |
| COPD | J4  J40  J41  J42  J43 | - | 0301  0302  0307 |
| Psychosis | F20-29 | - | 0402 |
| Depression | F32-33 | - | 0403 |
| **Post MI medications** |  |  |  |
| Antiplatelet | - | - | 0209  0204000AC |
| Lipid lowering medications | - | - | 0212 |
| ACEi/ARB | - | - | 0205051  0205052  0206020Z0 |
| BB/CCB | - | - | 0204  0206020C0  0206020T0 |
