# Supplementary figures and images for: Association of sociodemographic factors and comorbidity with non-receipt of medications for secondary prevention: a cohort study of 12,204 myocardial infarction survivors
Source: BMC Med. 2025 Jul 1;23:381. doi: 10.1186/s12916-025-04160-5 (PMC12219715; doi:10.1186/s12916-025-04160-5)

Supplementary Figure 1. Causal assumption for this study


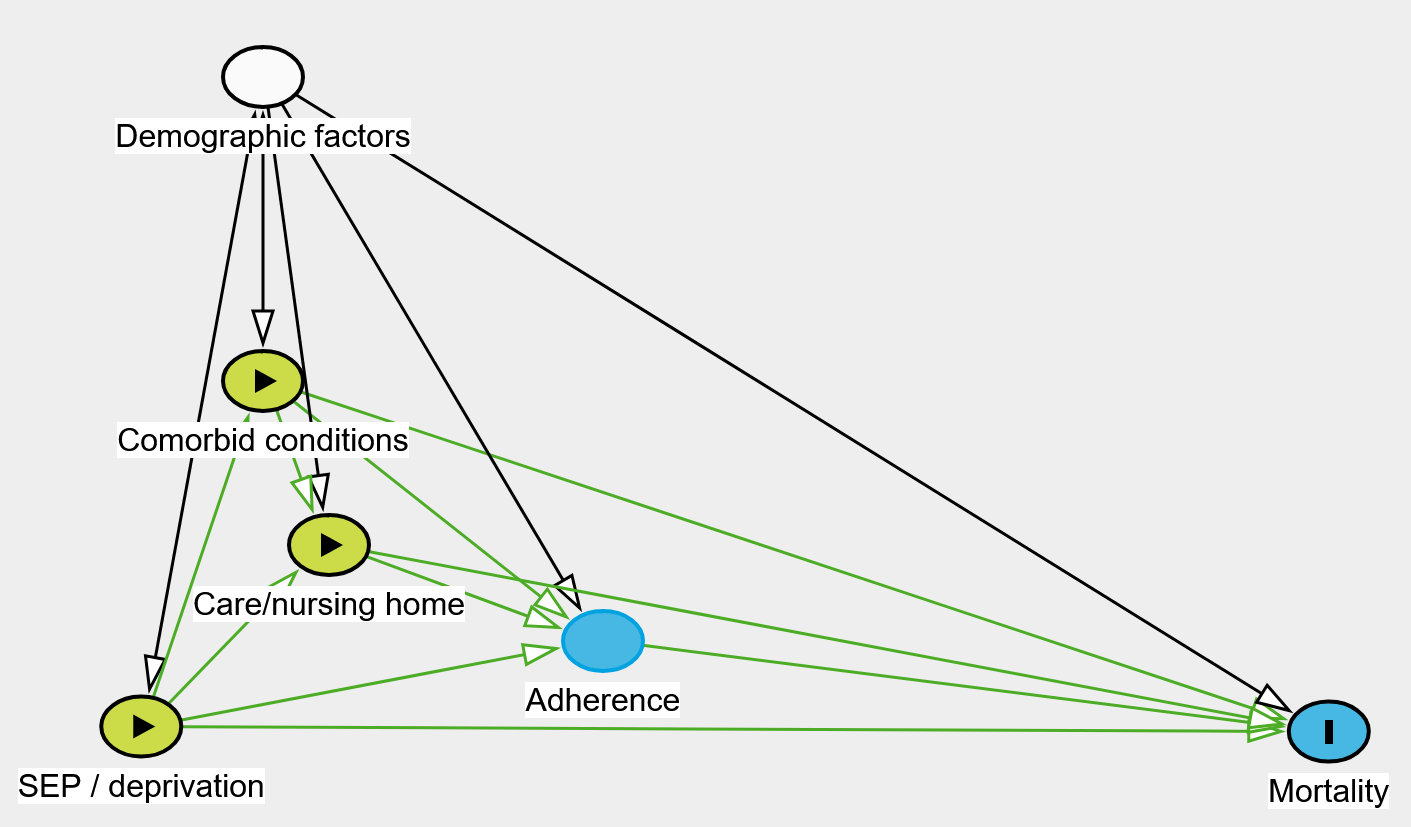

Supplement: Supplementary file 2 — Additional file 2: Figure S1 – Causal assumptions of this study. [file 12916_2025_4160_MOESM2_ESM.docx]
